# Enhanced Nanozymatic Activity on Rough Surfaces for H_2_O_2_ and Tetracycline Detection

**DOI:** 10.3390/bios14020106

**Published:** 2024-02-17

**Authors:** Tawfiq Alsulami, Abdulhakeem Alzahrani

**Affiliations:** Department of Food Science & Nutrition, College of Food and Agricultural Sciences, King Saud University, Riyadh 11451, Saudi Arabia

**Keywords:** black phosphorus, spiky gold nanoparticle, H_2_O_2_, tetracyclines, nanozyme

## Abstract

The needless use of tetracyclines (TCs) in foodstuffs is a huge health concern in low- and middle-income and Arab countries. Herein, a sensitive and faster monitoring system for H_2_O_2_ and TCs is proposed, utilizing the large surface-to-volume ratio of a non-spherical gold nanoparticle/black phosphorus nanocomposite (BP-nsAu NPs) for the first time. BP-nsAu NPs were synthesized through a single-step method that presented nanozymatic activity through 3,3′,5,5′-Tetramethylbenzidine (TMB) oxidation while H_2_O_2_ was present and obeyed the Michaelis–Menten equation. The nanozymatic activity of the BP-nsAu NPs was enhanced 12-fold and their detection time was decreased 83-fold compared to conventional nanozymatic reactions. The proposed method enabled us to quantify H_2_O_2_ with a limit of detection (LOD) value of 60 nM. Moreover, target-specific aptamer-conjugated BP-nsAu NPs helped us detect TCs with an LOD value of 90 nM. The present strategy provides a proficient route for low-level TC monitoring in real samples.

## 1. Introduction

Nanozymes (artificial nano-scale enzymes) have been receiving a lot of research focus recently because of their increased catalytic activity, higher stability compared to biological enzymes, ease of preparation and purification, and greater cost efficiency. Our ability to prepare nanomaterials with enzyme-like activity has improved; their nanozymatic performance can be enhanced through surface modification using ligands or by changing their chemical compositions, and they can be utilized in different applications [1,2,3,4,5,6,7,8,9]. In recent years, researchers have synthesized different nanomaterials with tunable sizes and surface charges to improve their nanozymatic activity [10]. It is well-established that the surface of a nanomaterial plays a significant role in enhancing its nanozymatic activity. However, the surface of a metallic nanozyme might undergo oxidation in contact with air, or a surrounding obstacle might deactivate its surface properties, which ultimately decreases the enzymatic performance of the nanomaterial. In this study, we considered several factors to improve nanozymatic activity. First of all, it is predictable that a hybrid nanozyme can show better nanozymatic activity than single nanomaterials. Second, free-radical-scavenging ligands containing nanomaterials are well-known to improve enzyme-like activity [11]. Thirdly, the catalysis reaction is much better on rough surfaces than smooth ones because of their higher surface activation energy [12]. However, no one has yet utilized this catalytic field in the context of nanozymatic activity, which might open a new window into colorimetric biosensor design. Lastly, and most importantly, accomplishing the integration of nanozymatic activity into electrochemical environments can accelerate the oxidation of TMB, which might improve sensitivity and shorten the detection time [13,14,15]. The authors of this paper have endeavored to consider all the above parameters to introduce a new electrochemical-assisted nanozymatic detection strategy that will be promising in bioassays in the future.

AuNPs have unwaveringly increased their attractiveness among different nanomaterials in recent few decades because of their beneficial features; for example, their optical nature, wide range of thermal stability, mechanical characteristics, chemical and electronic behaviors, and enzyme-like activity [16,17]. The remarkable biocompatibility of AuNPs allows for their superficial and steady pairing with several other materials to produce innovative nanohybridized materials with more than one property that will extend their application into a different field. Most notably, the tunable surface morphology of Au NPs can improve their performance in different applications, i.e., fluorescence biosensing and SERS-based detection. Moreover, thiolated molecules can be connected to Au NPs’ surfaces without extra chemicals or efforts, which makes them a favorable nanomaterial in the biosensing field. Also, the size of Au NPs is easy to tune, and smaller-sized Au NPs have better nanozymatic activity [10]. However, the size- and surface-ligand-dependent nanozymatic activity of Au NPs has been reported, but studies on the correlation of Au NPs’ shape to their enzyme-like activity have not yet been discussed. 

The popularity of two-dimensional (2D) nanomaterials among researchers, as well as companies, is increasing due to several outstanding properties [18,19,20]. BP, a two-dimensional material, has drawn tremendous interest since 2014 because of its spectacular carrier mobility, wide absorption range, and tunable bandgap, which can be adjusted using its thickness. However, its unbonded lone pairs of phosphorus atoms can easily react with oxygen, and BP undergoes oxidization and degradation when it comes into contact with air, which limits its application in biosensor development [21,22]. To improve its stability, common approaches are physical etching and chemical modification. Compared to the physical method, the chemical method is easier and more cost-effective. Hence, the present study utilized the chemical method to prepare a nanocomposite structure of BP and Au NPs to enhance the solubility as well as the stability of BP in an aqueous solution. 

The precise and fast measurement of H_2_O_2_ is vital for several diagnostic areas since H_2_O_2_ is a byproduct of several reactions [23]. By discovering a rapid, specific, and sensitive detection method for H_2_O_2_ in various conditions, scientists would be able to understand biological processes and cellular signaling. H_2_O_2_ is a common chemical used in water treatment and many chemical syntheses. Moreover, residues of H_2_O_2_ may be observed as end products of sterilization, pasteurization, and packaging. The ingestion or inhalation of H_2_O_2_ over a certain level is harmful for human health. Hence, the monitoring of H_2_O_2_ levels is essential for health and environmental safety. 

TCs are antibiotics widely used in the veterinary field to prevent animal diseases or to promote animal growth for extra economic benefits. Nonetheless, the overuse of TCs on agri-farms could incur several health impacts for consumers [3]. In particular, the excess addition of TCs to milk products in Arab countries may lead to congenital anomalies, mutagenic effects, and bone marrow dysfunction [24]. Considering the purchaser’s health, maximum residue levels of 100 ng mL^−1^ for TCs in milk samples have been suggested by the Codex Alimentarius regulatory agency [25]. Hence, a feasible and appropriate TC detection method is urgently required to monitor their unnecessary use in various fields. At present, TC detection methods mainly rely on using sophisticated machines such as capillary electrophoresis, mass spectrometry, and high-performance liquid chromatography technologies. Each of these machines needs an expert person to operate it, the detection time is longer, and the overall cost is higher. Compared to these techniques, colorimetric detection techniques offer a cheaper way to monitor H_2_O_2_ and better applicability, even having instrument-free detection strategies. 

In this study, tunable-shape Au NPs were attached to the surface of BP, and the nanozymatic activity of BP-Au NPs with varying shapes of Au NPs was then investigated using a conventional method and an integrated electrochemical method. The discovery of enhanced nanozymatic performance in BP-nsAu NPs was applied to monitor H_2_O_2_ and TCs in a complex matrix. 

## 2. Materials and Methods

### 2.1. Materials

Gold (III) chloride trihydrate (HAuCl_4_⋅3H_2_O), 30% *w*/*w* hydrogen peroxide (H_2_O_2_), TMB, hydroquinone, glutathione disulfide tetracyclines, and dimethyl sulfoxide (DMSO) were bought from Nanochemazone, Edmonton, AB, Canada. The tetracycline aptamer (5′-Fc-CCCCCGGCAGGCCACGGCTTGGGTTGGTCCCACTGCGCGT-(CH_2_)_3_-SH-3′) was collected from Integrated DNA Technologies, USA. 

### 2.2. Instruments

UV−visible spectra were collected using a BioTek spectrophotometer (Synergy H1, USA). Images of the nanomaterials were taken using a transmission electron microscope (FEI Titan, 80–300 LB, Hillsboro, OR, USA). An Agilent Cary 630 FTIR spectrometer (Santa Clara, CA, USA) was used for Fourier Transform Infrared (FTIR) measurements. Raman spectra were obtained using Raman spectroscopy (NRS-7100, JASCO Corporation, Tokyo, Japan).

### 2.3. Electrochemical Measurements

The electrochemical experiments were performed using a PalmSens4 potentiostat (EmStatMUX8-R2). Zensor screen-printed electrodes (Nanochemazone, Edmonton, AB, Canada) and PBS as an electrolyte were used in every electrochemical experiment.

### 2.4. Preparation of BP–Nonspherical Au NP Hybrid Structure

To prepare the nanohybrid structure of BP-nsAuNPs, 1 mg of BP was added to a microtube containing 1 mL of DI water (the total concentration was 1 mgmL^−1^) and gently refluxed at 100 °C for 10 min. Then, 100 µL of an aqueous solution of HAuCl_4_ (0.2 mM) was added to the above solution, and refluxing continued for 30 min. At this stage, gold ions (Au^3+^) will adsorb on the surface of the BP through electrostatic force. From another microtube, 0.05 M (10 µL) of hydroquinone and 0.5 mg of glutathione disulfide dissolved in DI water (500 µL) were transferred into a BP-Au^3+^ solution. The solution’s initial light-yellow color turned purple within 10 min, indicating the formation of AuNPs. After 30 min of the reaction, the solution was centrifuged at 15,000 rpm for 20 min, and the nanohybrid structure of the BP-AuNPs was collected, dried in oven, and stored at 4 °C for further analysis.

To modify the surfaces of the BP-nsAu NPs, a 1 mg/mL aqueous solution of BP-nsAu NPs was mixed with a 0.1 M solution of NaSCN for 1 h. At this stage, the SCN groups will bind with the Au NPs of the BP-nsAu NP composites. After washing them several times, the thiocyanide-modified BP-nsAu NP (SCN/BP-nsAu NP) composites were dried and kept at 4 °C.

### 2.5. Preparation of BP–Spherical Au NP Hybrid Structure

To prepare the nanohybrid structure of BP–spherical AuNPs, 1 mg of BP was added to a microtube containing 1 mL of DI water (the total concentration was 1 mgmL^−1^) and gently refluxed at 100 °C for 10 min. Then, 100 µL of an aqueous solution of HAuCl_4_ (0.2 mM) was added to the above solution, which continued refluxing for 30 min. From another microtube, 0.05 M (100 µL) of sodium borohydride was transferred to a BP-Au^3+^ solution. The initial light-yellow solution color of the resultant BP-Au^3+^ turned purple within 10 min, indicating the formation of AuNPs. After 30 min of the reaction, the solution was centrifuged at 15,000 rpm for 20 min, and the nanohybrid structure of BP-AuNPs was collected, dried in oven, and stored at 4 °C for further analysis. 

### 2.6. Conjugation of Aptamer with SCN/BP-nsAu NPs

The thiolated aptamer was conjugated with Au NPs of SCN/BP-nsAu NPs through a thiol–gold interaction. At first, the tetracycline aptamer was suspended in DI water (1 mL) and heated at 95 °C for 3 min. Then, it was cooled down to room temperature to generate single-stranded structures. Next, 200 µL of aptamer solution was added to 800 µL of the SCN/BP-nsAu NP solution (1 mgmL^−1^), and the solution was mixed with stirring for 2 h at room temperature. Then, the conjugated part was separated through centrifugation (5000 rpm, 30 min) and redispersed in 1 mL of MilliQ water. The purification was repeated three times, and the solution was stored at 4 °C using PBS media (pH 7.4).

### 2.7. The Optimization of Reaction Conditions 

Several concentrated H_2_O_2_ samples were prepared from 30% *w*/*w* H_2_O_2_ using DI water. To prepare different solutions of TMB, at first, 10 mg of TMB was dissolved in 1 mL of DMSO. Then, a series of dilutions was prepared using DI water. To determine the best nanozymatic activity, the reaction was performed at different pH levels.

### 2.8. Kinetic Parameter Study of BP-Au NPs 

To examine the kinetic behavior of SCN/BP-Au NPs, the effects on the absorbance values (at 660 nm) of varying concentrations of TMB and H_2_O_2_ over the course of 30 min were measured at 2 min intervals. While the concentration of TMB varied from 0–20 mM, the concentration of H_2_O_2_ was fixed at 0.05 mM. Similarly, while the absorbance values of different concentrations of H_2_O_2_ (0–24 mM) were measured, the TMB concentration was fixed at 10 mM. After converting the absorbance values to initial velocities using a modified version of Beer–Lambert’s law, Michaelis–Menten and Lineweaver–Burk plots were constructed to determine the *K*_m_ and *V*_max_ kinetic parameters. 

### 2.9. DPPH Test

A DPPH test was performed to monitor the free-radical-scavenging capability of the synthesized BP-Au NPs in the present study. At first, different concentrated solutions of BP-Au NPs were combined with a DPPH (100 µM) solution and stored in a dark atmosphere. The absorbances of different solutions were checked after 30 min. The following equation was used to calculate the scavenging activity:

Scavenging activity (%) = (*A*a − *A*b)/*A*a (1)

where *A*a and *A*b represent the absorbances of the DPPH free radicals in the absence and presence of the BP-Au NPs, respectively.

### 2.10. Terephthalic Acid (TA) Test

The hydroxyl (•OH)-radical-formation ability of the BP-Au NPs was monitored through the TA test. To do so, 1 mM (50 µL) of an aqueous solution of TA was mixed with the BP-Au NP solution (50 µL) and kept at room temperature for 10 min. Then, an H_2_O_2_ solution (10 mM, 50 µL) was added to it and after 30 min, the fluorescence intensity of the reaction product was recorded using a fluorescence spectrophotometer. 

### 2.11. H_2_O_2_ Detection Using BP-Au NPs

H_2_O_2_ detection was conducted through colorimetric detection. Briefly, a 1:1 volume solution (20 µL of each) of 10 mM TMB and SCN/BP-nsAuNPs (1 mgmL^−1^) was mixed on the working electrode zone area of a screen-printed electrode. Then, a varying concentration of H_2_O_2_ (0–100 µM, 50 µL) was added to the above solution, and cyclic voltammetry (CV) was carried out in voltage range of −1.0–1.0 V with a 0.1 V/s scan rate [26]. At this stage, the blue color of oxidized TMB appeared on the working electrode area of the screen-printed electrode (SPE) which was transferred to a 96-well plate (50 µL for each sample). Then, the detection sensitivity was determined by the characteristic blue color, and the intensities were recorded using a UV–vis spectrometer. 

### 2.12. Tetracycline Detection Using BP-Au NPs

A similar experiment was conducted for aptamer-based TC detection through the nanozymatic activity of SCN/BP-nsAu NPs. For example, 20 µL of BP-AuNPs, 20 µL of TMB, 20 µL of H_2_O_2_, and 20 µL of TCs were mixed on the working electrode zone area of a screen-printed electrode, and CV was carried out in a voltage range of −1.0–1.0 V with a 0.1 V/s scan rate to develop a deep blue color. The intensity of the developed color was recorded using a UV–vis spectrometer. 

The present study works at a ramped voltage from −1 V to 1 V with a scan rate of 0.1 V/s for both H_2_O_2_ and TC detection. Here, the main goal was to improve the color or oxidation of TMB in an electrochemical setting. Hence, a flow of current was required to develop a deeper blue color due to the oxidation of TMB, enhance the sensitivity, and decrease the detection time. The optimization of electrochemical parameters was not required or performed here since the present work does not present any electrochemical sensing experiments.

## 3. Results

**Scheme 1:**
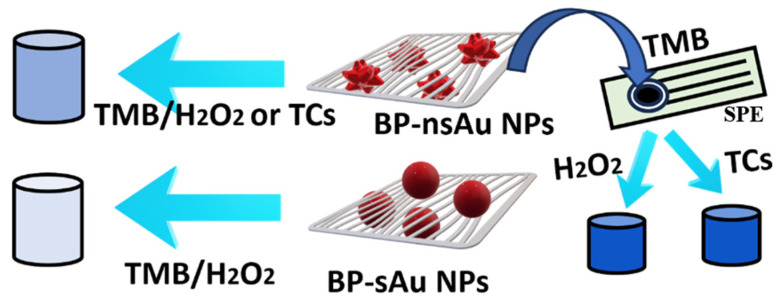
Schematic presentation of the proposed sensing strategy.

The groundbreaking discovery of the enzyme-like nature of nanomaterials made a bridge connecting biotechnology and nanotechnology. The invention of new nanozymes with improved activity is increasing day by day. Furthermore, surface modifications to introduce or enhance nanozymatic activity also increase, for example, the introduction of a free radical scavenger on the surfaces of nanomaterials could help boost the nanozymatic activity since all reactions will occur on the surfaces of nanomaterials [3,4]. Here, a nanomaterial works as a catalyst for the oxidation reaction. The greater oxidation of a chromogenic substrate means a deeper-colored solution and enhances the sensitivity of detection. Moreover, the faster the oxidation process, the shorter the detection time. In this article, SCN was used as a radical scavenger, and the spiky structure of the Au NPs was used to make more space for nanozyme–substrate reactions. Lastly and most importantly, the entire reaction was performed under an electrochemical potential to significantly augment the oxidation process, time and detection sensitivity (Scheme 1). Considering all the parameters in the present sensing system brings the proposed idea onto a novel platform for various sensing applications in science and engineering. In particular, the proposed method will be promising in replacing the conventional ELISA-to-Electro-assisted ELISA in different applications.

**Figure 1:**
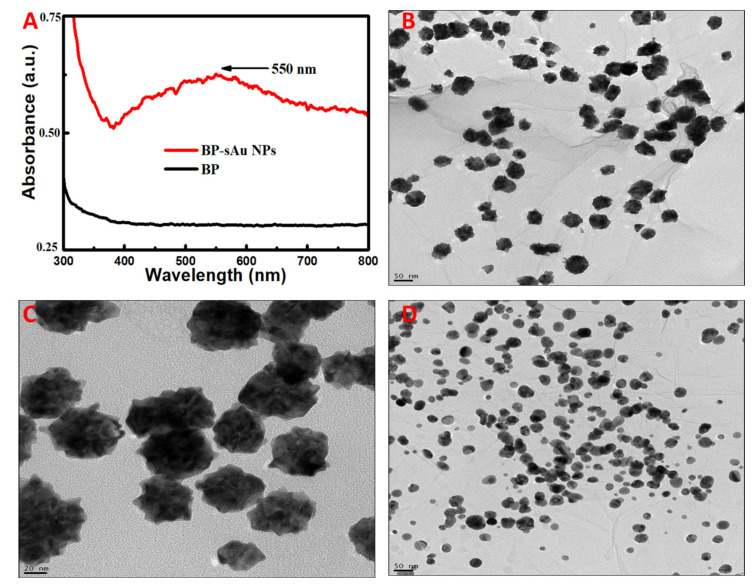
Characterization of BP-Au NPs: (**A**) absorbance spectra of composite; (**B**) far-view TEM image of BP-sAu NPs; (**C**) close-view TEM image of BP-sAu NPs; and (**D**) TEM image of BP-Au NPs.

At first, the absorbance spectra of pristine BP and BP-Au NPs were measured using UV–visible spectroscopy. As shown in Figure 1A, the pristine BP showed a broad absorbance spectrum from 400–700 nm. However, a distinguishable plasmonic peak appeared in the BP-sAu NP composites. In both cases, very similar plasmonic peak positions (at around 540 nm) and optical densities appeared, indicating that the sizes and concentrations of the Au NPs on the BP surfaces were very similar. The nanozymatic activity of nanomaterials is strongly influenced by the nanoparticles’ size and concentration. Hence, it was crucial to keep both factors similar to compare the effects of shape on nanozymatic activity. Then, the shape of the Au NPs on the BP surface was investigated through transmission electron microscopy. As shown in Figure 1B,C, the average size of non-spherical Au NPs was 50 nm, with some spikes on the surface. Similarly, the size of spherical Au NPs was 45 nm. It is well -known that a rough surface has several small peaks and valleys, which means more surface area. The catalytic reaction is directly proportional to the surface area of a nanomaterial. Unfortunately, no one has yet discovered the effect of a rough nanomaterials surface on its nanozymatic activity. Hence, this study focuses on unveiling this unknown science. The synthesized nanocomposite has shown a wide range of pH stability (2–9 pH) and reproducibility. 

The Raman spectroscopy of pristine BP and BP-Au nanocomposites is presented in Figure S1. The Raman data analysis revealed that three Raman peaks appeared at 365, 432, and 464 cm^−1^ in all cases attributed to the A^1^g, B_2g_, and A^2^g vibrational modes, respectively. Moreover, the Raman study confirmed that the phase (orthorhombic) nature of the pristine BP was maintained even after Au NPs attached to its surface. A slightly shifted and weakened Raman peak of nanocomposites compared to pristine BP was observed due to the partial interruption of intra-layer phosphorus bonding. The as-synthesized nanocomposite showed better solubility in an aqueous solution (Figure S2).

**Figure 2:**
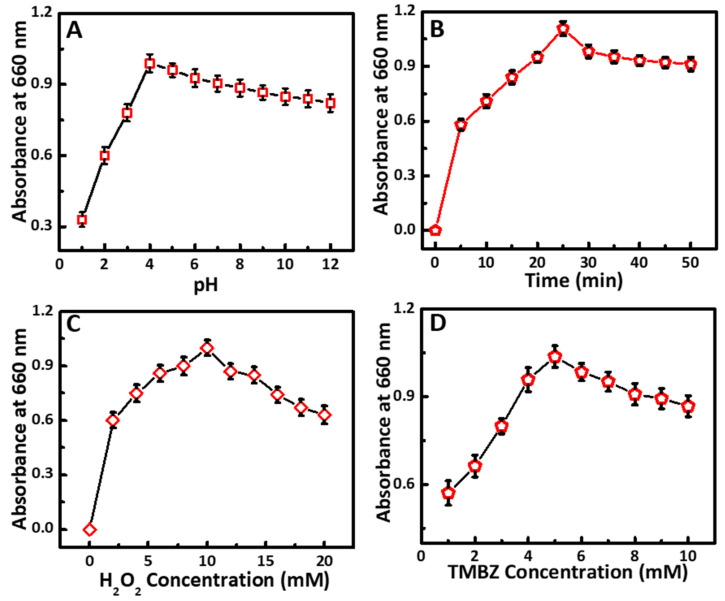
Optimization of (**A**) pH; (**B**) reaction time; (**C**) concentration of H_2_O_2_; and (**D**) concentration of TMBZ.

Before investigating the nanozymatic activity of BP-Au NPs, several parameters were optimized, i.e., the pH of the solution, the reaction time, the TMB concentration, and H_2_O_2_ concentration (Figure 2). The nanozymatic activity of nanocomposite was stronger at a pH of around 4; this was used for the rest of the experiments (Figure 2A). Nanozymatic activity decreased at higher pH values because of the instability of nanomaterials, in particular, Au NPs lose their surface functionality at higher pH levels, start aggregating and lose the activity. Similarly, the present composite showed better nanozymatic activity at room temperature due to stability issues at higher temperatures. For example, the zeta potential of the nanocomposite decreased drastically at a higher temperature, which promotes the aggregation and agglomeration of the AuNPs (Table S1). Most probably, the electrostatic repulsion might be more dominant over the attractive force at room temperature compared to higher temperatures that help Au NPs avoid the aggregation and keep their activity. The reaction time to reach the highest absorbance value was 25 min at room temperature (Figure 2B). The optimized TMB and H_2_O_2_ concentrations were 5 mM and 10 mM, respectively (Figure 2C,D).

The kinetic parameters of the BP-nsAu NPs were analyzed to obtain the Michealis–Menten constant (*K*_m_) and the maximum velocity (*V*_max_). The K_m_ value and affinity between the substrate and enzyme are inversely related, for example, the lower the value of K_m_, the higher the nanozyme–substrate affinity. The calculated K_m_ values for TMB and H_2_O_2_ were 4.9 mM and 2.24 mM, respectively. The *V*_max_ values for TMB and H_2_O_2_ were 4.8 × 10^−7^ mM/s and 4.093 × 10^−3^ mM/s, respectively. A comparison study of kinetic parameters and the published articles are presented on Tables S2 and S3. The obtained values of kinetic parameters using BP-nsAuNPs prove their high competency with the reported values and reveal that the synthesized nanozyme and proposed assay strategy is promising for colorimetric biosensing applications. 

**Figure 3:**
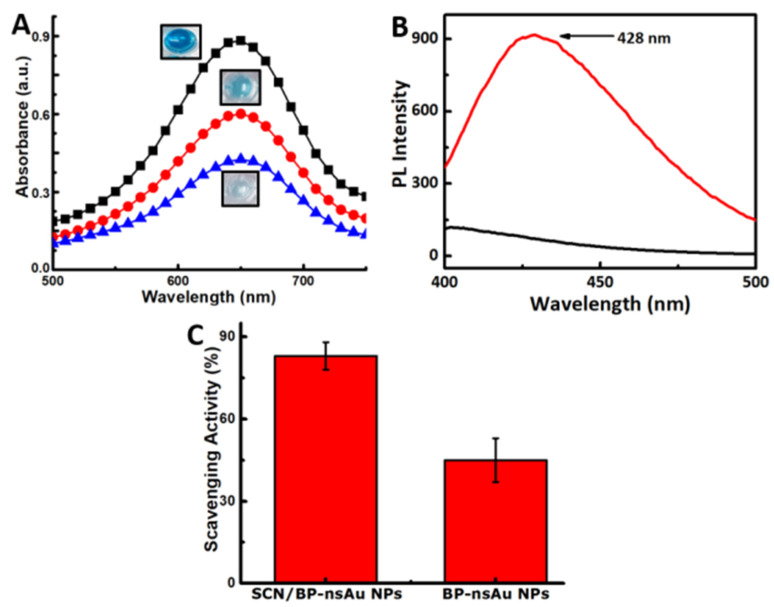
(**A**) Comparison study of the nanozymatic activity of pristine BP (blue line); BP-sAuNPs (red line), and BP-nsAu NPs (black line); (**B**) TA test result (red: in the presence of BP-nsAu, TA, and H_2_O_2_ and black: BP-nsAu NPs and TA) and (**C**) DPPH results of BP-nsAu NPs and SCN-modified BP-nsAu NPs.

Then, the nanozymatic activity of pristine BP, BP-sAuNPs, and BP-nsAu NPs was measured at room temperature. As shown in Figure 3, the BP-nsAu NPs showed greater nanozymatic performance compared to pristine BP and the BP-sAu NPs. Here, the larger surface area of the BP-nsAu NPs composite creates more room for a substrate (H_2_O_2_) to react and produce a deeper color of oxidized TMB. Moreover, TA and DPPH tests were performed to check the generation and scavenging of free radical by BP-nsAu NPs. As shown is Figure 3B, a strong fluorescence peak was monitored in the presence of BP-nsAu NPs, TA, and H_2_O_2_ indicated the reaction of TA and hydroxyl radicals produces a fluorescent hydroxyl terephthalic acid product. However, no fluorescent peak was detected in the absence of H_2_O_2_. Hence, it is clear that a hydroxyl radical was formed in the presence of BP-nsAu NPs, TA and H_2_O_2_. The hydroxyl radicals provide a meaningful contribution to the nanozymatic activity of nanomaterials since free radicals initiate the oxidation reaction of TMB which, accordingly, creates the blue color solution. Moreover, the radical scavenging activity of the nanocomposite increased significantly after the surface modification with SCN groups, indicating that the activity of the SCN group retained even in the composite structure (Figure 3C).

**Figure 4:**
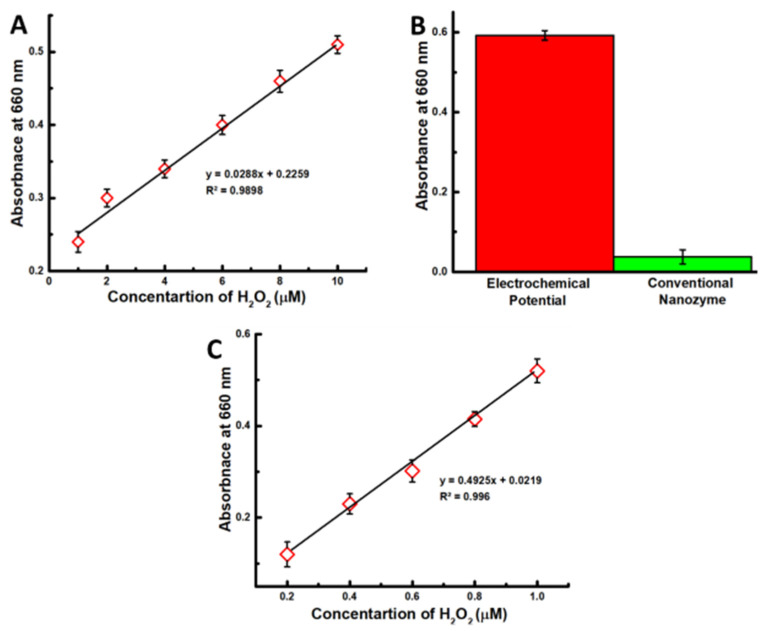
(**A**) Nanozymatic detection of H_2_O_2_ using a conventional method; (**B**) absorbance (at 660 nm) using electrochemical potential and the conventional method and (**C**) nanozymatic detection of H_2_O_2_ using electrochemical potential.

Then, a calibration curve of H_2_O_2_ was constructed using BP-nsAu NPs. As shown in Figure 4A, a linear curve in the range of 10–1 µM was achieved, and the calculated LOD was 0.4 µM. However, the detection time was around 25 min, which is a big drawback of this method. To overcome this problem, the same experiment was performed on screen-printed electrodes under electrochemical potential. While the nanozymatic activity assessment of the BP-nsAu NPs, H_2_O_2_, and TMB mixture solution was performed for both conventional and electrochemical potential, a 12-fold enhancement of optical density was observed during the electrochemical process compared to the conventional nanozymatic process (Figure 4B). Moreover, the reaction time to reach the highest optical density was only 18 s, indicating that the oxidation reaction was 83 times faster under electrochemical potential compared to a conventional nanozymatic assay (25 min). Most probably, the oxidation potential of TMB was reduced extremely in an electrochemical environment and accelerated the oxidation process of TMB significantly. Here, the nanocomposite structure of BP-nsAu NPs acts as an electrocatalyst, and a large amount of TMB is oxidized within a short period of time. To investigate the electrocatalytic behaviour of the nanocomposite, an oxygen evolution reaction (OER) involving simple GCE, BP-nsAu NPs, and SCN/BP-nsAu NPs was performed.

As shown in Figure S3, the values of the Tafel slope for bare GCE, BP-nsAu NPs, and SCN/BP-nsAu NPs were 3.65 mV/dec, 2.38 mV/dec and 1.18 mV/dec, respectively, revealing that the SCN-modified BP-nsAu NPs have the highest OER activity and promising electrocatalytic activity.

The present findings were utilized for the detection of H_2_O_2,_ and the results are presented in Figure 4B. The detection range was 0.2–1 µM, and the calculated LOD was 0.06 µM (based on the standard deviation method). The electrochemical potential-based nanozymatic activity showed a significant sensitivity compared to the recent published articles (Table S4).

**Figure 5:**
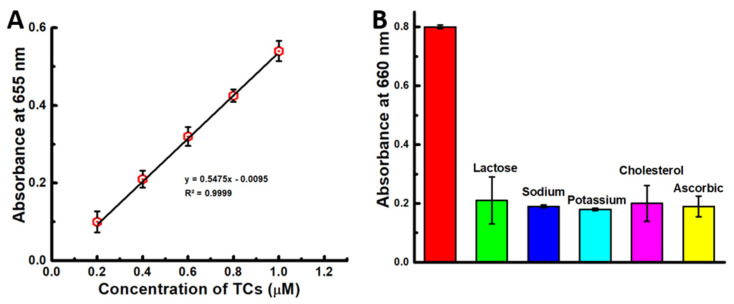
(**A**) Nanozymatic detection of TCs under electrochemical potential; (**B**) selectivity of TC detection.

The electrochemical-assisted enhanced nanozymatic detection of TCs using aptamer-modified SCN/BP-nsAu NPs was presented in Figure 5A. The detection range was 0.2–1 µM, and the calculated LOD was 0.09 µM. Herein, assuming that aptamer-modified SCN/BP-nsAu NPs played a significant role to bring nanomaterials closer to each other while TCs are added and we creäte more radicals, accelerating the nanozymatic reaction [27]. The present method showed meaningful sensitivity compared to other recently published articles (Table S5). Though the published articles in Figure 5 reported a wide concentration range of detection, our aim was to check the sensitivity at a lower concentration with a shorter interval (0.2 µM), which might be helpful in distinguishing different concentrations at the lower end. As shown in Table S5, the proposed assay strategy would be promising to monitor lower concentrations of TCs and might be a stronger candidate for replacing the current ELISA technique. 

The specificity of the TC detection was determined in the presence of common interfering substrates (Figure 5B). Optical density was much higher in the presence of TCs compared to the interfering substrates, indicating that the proposed method is highly selective to the target analyte. Even the presence of ascorbic acid did not affect the detection of TCs.

The real-life applicability of the proposed method was measured using a spiked milk sample. The recovery results in milk samples have been presented in Table S6, indicating that the sensing of TCs in spiked milk samples has a recovery value of 97.5% to 108.30% and an RSD value was less than 4.0%. These results indicate that the proposed assay was highly accurate and reliable for TC detection in real-world samples. Moreover, the stability of the nanocomposite was found to be up to six months in different media (Table S7), and the shelf-life of the biosensor was up to 100 days, revealing the stability of the proposed biosensor (Figure S5).

## 4. Conclusions

The present study focused on the enhancement of nanozymatic activity and shortening the analyte detection time by integrating electrochemical potential into conventional nanozyme-based detection. At first, a nanocomposite structure of BP-nsAu NPs with free-radical-scavenging ligands on the surface was prepared, and the nanozymatic activity was examined. The results revealed that the attachment of free-radical-scavenging ligands, the shape of nanomaterials and the reaction of the nanozyme under electrochemical settings has significant role in enhancing optical density and decreasing detection time. Here, non-spherical shaped NPs gives more room for nanozyme-substrate reaction and the electrochemical force accelerates the oxidation of TMB. The present method enabled us to detect H_2_O_2_ and TCs with LOD values of 60 nM, and 90 nM, respectively. Though a detailed study of electrochemical nature of the composite is still unknown, the present study will introduce a new detection method in a shorter time and a possible way to integrate electrochemical settings into a conventional ELISA method for different applications.

## Data Availability

The data presented in this study are available upon request from the corresponding author.

## Supplementary Materials

The following supporting information can be downloaded at https://www.mdpi.com/article/10.3390/bios14020106/s1, Figures S1: Raman spectra of pristine BP and BP-Au NPs; Figure S2: Solubility of BP-Au NPs before (left) and after (right) composite preparation; Figure S3: Electrochemical characterization of BP-nsAu NPs through OER; Figure S4: Shelf-life of biosensor; Table S1: Zeta potential measurement with different temperature; Table S2: Comparison study of K_m_ and V_max_ values for the present work with others reported using H_2_O_2_ as the substrate; Table S3: Comparison study of *K*_m_ and *V*_max_ values for the present work with others reported using TMB as the substrate; Table S4: A comparison study of nanozyme-based H_2_O_2_ detection; Table S5: A comparison study of nanozyme-based TC detection; Table S6: Precision (relative standard deviation, RSD%) and recovery study of the TCs from milk samples; Table S7: Stability of nanocomposite. The references [28,29,30,31,32,33,34,35,36,37,38,39,40,41,42,43,44,45,46,47,48,49,50,51,52,53] are cited in the supplementary materials.
