# Supplementary material for: Enhanced Nanozymatic Activity on Rough Surfaces for H2O2 and Tetracycline Detection
Source: Biosensors (Basel). 2024 Feb 17;14(2):106. doi: 10.3390/bios14020106 (PMC10886513; doi:10.3390/bios14020106)
Supplement: Supplementary file 1 [file biosensors-14-00106-s001.zip › biosensors-2841631-supplementary.pdf]

Enhanced Nanozymatic Activity on Rough Surfaces for H_2O_2 and Tetracycline Detection

Tawfiq Alsulami and Abdulhakeem Alzahrani *

Department of Food Science & Nutrition, College of Food and Agricultural Sciences, King Saud University, Riyadh 11451, Saudi Arabia
* Correspondence: aabdulhakeem@ksu.edu.sa

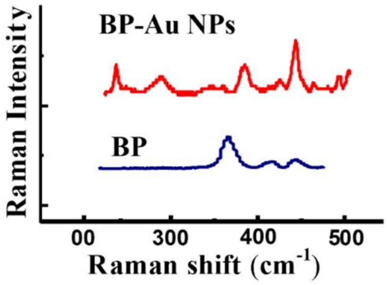

Figure S1. Raman spectra of pristine BP and of BP-Au NPs.

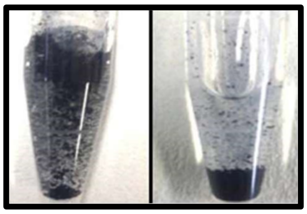

Figure S2. Solubility of BP-Au NPs: before (left) and after (right) composite preparation.

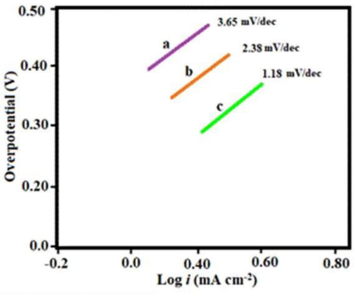

Figure S3. Electrochemical characterization of BP-nsAu NPs through OER.

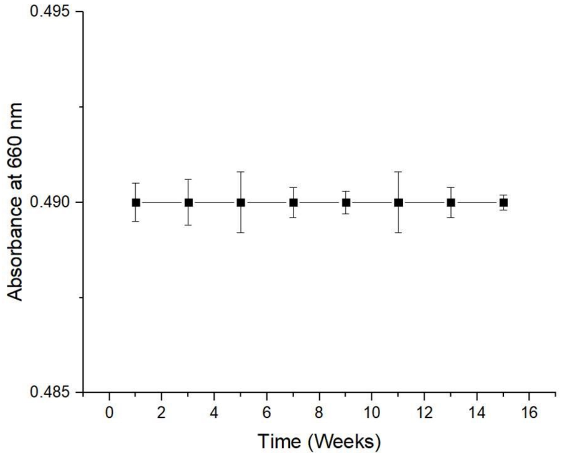

Figure S4. Shelf-life of biosensor.

Table S1. Zeta potential measurement with different temperature.

**Temperature (°C)**	**Zeta Potential (mV)**
25	-31.2
30	-25.3
35	-13.8

Table S2. Comparison study of K_m and V_{max} values for present work with others reported using H_2O_2 as the substrate.

	**H_2O_2 Substra**	**te**	
Nanomaterial	K_m (mM)	V_{max} (mM/s)	Ref.
HRP	3.7	8.71×10^{-5}	1
Brominated Graphene (GBR)	10.98	3.60×10^{-7}	2
$Cu_2(OH)_3Cl$-CeO_2	11.61	8.15×10^{-5}	3
V_2O_5 NPs	26.47	-	4
AuNP	33	6.10×10^{-5}	5
Fe_3O_4-COOH	65.33	14.02	6
AuFt	199.4	9.34×10^{-5}	7
PBMNPs3	323.6	1.17×10^{-3}	8
$FesO_4$@Pt	702.6	7.13×10^{-4}	9
$FesO_4$	1175.3	2.40×10^{-4}	
BP-nsAu NPs	2.24	4.093×10^{-3}	This work.

Table S3. Comparison study of K_m and V_{max} values for present work with others reported using TMB as the substrate.

	**TMB Substrat**	**e**	
Nanomaterial	K_m (mM)	V_{max} (mM/s)	Ref.
Gold Nanosphere (Au NS)	1.303	1.09×10^{-5}	10
WS_2	1.83	4.31×10^{-5}	11
CoOOH	2.02	4.74×10^{-5}	12
CuZnFeS	2.2	3.90×10^{-4}	13
Nanoceria	3.8	7.00×10^{-4}	14
Ce-Fe_3O_4 MNPs	6.942	5.00×10^{-4}	15
N-GQDs	11.9	3.80×10^{-6}	16
$Cu_2(OH)_3Cl$-CeO_2	12.36	1.06×10^{-4}	17
MoS2-Pt74Ag26	25.71	7.29×10^{-5}	18

BP-nsAu NPs	4.9	4.8×10^{-7}	This work.

Table S4. A comparison study of nanozyme-based H_2O_2 detection.

Material	Method	Linear Range (mM)	LOD (mM)	Ref
VO_2 nanorods	Colorimetric	0.488 – 15.625	0.41	19
Au@PNIPAm	Colorimetric	3 - 15	2.43	20
Pd-Pt-Ir	Colorimetric	3.9 – 62.5	3.6	21
Au-Ag-Pt	Colorimetric	0.05 - 1	0.054	22
Ni-MOF	Fluorescent	0.1 - 20	0.05	1
BP-nsAu NPs	Colorimetric	0.0002–0.001	0.00024	This work

Table S5. A comparison study of nanozyme-based TCs detection.

Nanozyme	Method	Linear Range	LOD	Ref
Au NCs	Colorimetric	1.5–30.0 μM	0.2 μM	23
Au NCs	Colorimetric	1-16 μM	0.046 μM	24
Mn_3O_4	Colorimetric	0.5–150 μM	0.1 μM	25
Fe_3O_4	Colorimetric	2–55 μM	0.4 μM,	26
Fe–N–C	Colorimetric	0.09–100 μM	0.062 μM	27
BP-nsAu NPs	Colorimetric	0.2-1 μM	0.09 μM	This work.

Table S6. Precision (relative standard deviation, RSD%) and recovery study of the TCs from milk samples.

Sample	Labelled (μM)	Found (μM)	Recovery (%)	RSD (%)	Coefficent of variation
1	10	09.70	97.00	3.21	0.35
2	08	08.30	103.75	2.68	0.21
3	06	06.50	108.30	3.59	0.19
4	04	03.90	97.50	3.72	0.24

Table S7. Stability of nanocomposite.

Types	No	Name	Zeta Potential (mV)	Stability (4ºC)
Buffers	1	PIPES	-12.5	Over 6 month
	2	MES	-10.5	Over 6 month
	3	TAPSO	-17.9	Over 7 month
	4	TAPS	-17.5	Over 4 month
	5	Bicine	-19.90	Over 8 month
	6	TES	-14.3	Over 7 month
	7	Triethanolamine	-12.0	Over 6 month
	8	Bis-Tris Methane	-12.3	Over 4 month
	9	Bis-Tris Propane	-17.2	Over 4 month
	10	PIPES Ses-quisodium	-11.2	Over 6 month
Chemicals	11	PSTT	-10.1	Over 6 month
	12	Sodium Meta-bisulfite	-15.7	Over 6 month

References

1. Guo, J.; Liu, Y.; Mu, Z.; Wu, S.; Wang, J.; Yang, Y.; Zhao, M.; Wang, Y. Label-Free Fluorescence Detection of Hydrogen Peroxide and Glucose Based on the Ni-MOF Nanozyme–Induced Self-Ligand Emission. Microchim. Acta 2022, 189 (6), 219. https://doi.org/10.1007/s00604-022-05313-6.
2. Gao, L.; Zhuang, J.; Nie, L.; Zhang, J.; Zhang, Y.; Gu, N.; Wang, T.; Feng, J.; Yang, D.; Perrett, S.; Yan, X. Intrinsic Peroxidase-like Activity of Ferromagnetic Nanoparticles. Nat. Nanotechnol. 2007, 2 (9), 577–583. https://doi.org/10.1038/nnano.2007.260.
3. Singh, S.; Mitra, K.; Singh, R.; Kumari, A.; Gupta, S. K. S.; Misra, N.; Maiti, P.; Ray, B. Colorimetric Detection of Hydrogen Peroxide and Glucose Using Brominated Graphene. Anal. Methods 2017, 9 (47), 6675–6681. https://doi.org/10.1039/C7AY02212C.
4. Song, Y.; Qu, K.; Zhao, C.; Ren, J.; Qu, X. Graphene Oxide: Intrinsic Peroxidase Catalytic Activity and Its Application to Glucose Detection. Adv. Mater. 2010, 22 (19), 2206–2210. https://doi.org/10.1002/adma.200903783.
5. Ezzatfar, R.; Dehghan, G.; Amini, M.; Khataee, A. Synthesis of Peroxidase-Like V2O5 Nanoparticles for Dye Removal from Aqueous Solutions. Top. Catal. 2022, 65 (5), 694–702. https://doi.org/10.1007/s11244-021-01523-z.
6. Liu, Y.; Wang, C.; Cai, N.; Long, S.; Yu, F. Negatively Charged Gold Nanoparticles as an Intrinsic Peroxidase Mimic and Their Applications in the Oxidation of Dopamine. J. Mater. Sci. 2014, 49 (20), 7143–7150. https://doi.org/10.1007/s10853-014-8422-x.
7. Bilalis, P.; Karagouni, E.; Toubanaki, D. K. Peroxidase-like Activity of Fe3O4 Nanoparticles and Fe3O4-Graphene Oxide Nanohybrids: Effect of the Amino- and Carboxyl-Surface Modifications on H2O2 Sensing. Appl. Organomet. Chem. 2022, 36 (9), e6803. https://doi.org/10.1002/aoc.6803.
8. Jiang, X.; Sun, C.; Guo, Y.; Nie, G.; Xu, L. Peroxidase-like Activity of Apoferritin Paired Gold Clusters for Glucose Detection. Biosens. Bioelectron. 2015, 64, 165–170. https://doi.org/10.1016/j.bios.2014.08.078.
9. Zhang, X.-Q.; Gong, S.-W.; Zhang, Y.; Yang, T.; Wang, C.-Y.; Gu, N. Prussian Blue Modified Iron Oxide Magnetic Nanoparticles and Their High Peroxidase-like Activity. J. Mater. Chem. 2010, 20, (24), 5110–5116. https://doi.org/10.1039/C0JM00174K.
10. Ma, M.; Xie, J.; Zhang, Y.; Chen, Z.; Gu, N. Fe3O4@Pt Nanoparticles with Enhanced Peroxidase-like Catalytic Activity. Mater. Lett. 2013, 105, 36–39. https://doi.org/10.1016/j.matlet.2013.04.020.
11. Ghosh, S.; Singh, P.; Roy, S.; Bhardwaj, K.; Jaiswal, A. Superior Peroxidase-Like Activity of Gold Nanorattles in Ultrasensitive H2O2 Sensing and Antioxidant Screening. ChemBioChem 2022, 23 (8), e202100691. https://doi.org/10.1002/cbic.202100691.
12. Lin, T.; Zhong, L.; Song, Z.; Guo, L.; Wu, H.; Guo, Q.; Chen, Y.; Fu, F.; Chen, G. Visual Detection of Blood Glucose Based on Peroxidase-like Activity of WS2 Nanosheets. Biosens. Bioelectron. 2014, 62, 302–307. https://doi.org/10.1016/j.bios.2014.07.001.
13. Wang, Y.-M.; Liu, J.-W.; Jiang, J.-H.; Zhong, W. Cobalt Oxyhydroxide Nanoflakes with Intrinsic Peroxidase Catalytic Activity and Their Application to Serum Glucose Detection. Anal. Bioanal. Chem. 2017, 409 (17), 4225–4232. https://doi.org/10.1007/s00216-017-0372-0.
14. Dalui, A.; Pradhan, B.; Thupakula, U.; Khan, A. H.; Kumar, G. S.; Ghosh, T.; Satpati, B.; Acharya, S. Insight into the Mechanism Revealing the Peroxidase Mimetic Catalytic Activity of Quaternary CuZnFeS Nanocrystals: Colorimetric Biosensing of Hydrogen Peroxide and Glucose. Nanoscale 2015, 7 (19), 9062–9074. https://doi.org/10.1039/C5NR01728A.
15. Asati, A.; Santra, S.; Kaittanis, C.; Nath, S.; Perez, J. M. Oxidase-Like Activity of PolymerCoated Cerium Oxide Nanoparticles. Angew. Chem. 2009, 121 (13), 2344–2348. https://doi.org/10.1002/ange.200805279.
16. Hosseini, M.; Sabet, F. S.; Khabbaz, H.; Aghazadeh, M.; Mizani, F.; Ganjali, M. R. Enhancement of the Peroxidase-like Activity of Cerium-Doped Ferrite Nanoparticles for Colorimetric Detection of H2O2 and Glucose. Anal. Methods 2017, 9 (23), 3519–3524. https://doi.org/10.1039/C7AY00750G.
17. Lin, L.; Song, X.; Chen, Y.; Rong, M.; Zhao, T.; Wang, Y.; Jiang, Y.; Chen, X. Intrinsic Peroxidase-like Catalytic Activity of Nitrogen-Doped Graphene Quantum Dots and Their Application in the Colorimetric Detection of H2O2 and Glucose. Anal. Chim. Acta 2015, 869, 89–95. https://doi.org/10.1016/j.aca.2015.02.024.
18. Cai, S.; Han, Q.; Qi, C.; Lian, Z.; Jia, X.; Yang, R.; Wang, C. Pt74Ag26 Nanoparticle-Decorated Ultrathin MoS2 Nanosheets as Novel Peroxidase Mimics for Highly Selective Colorimetric Detection of H2O2 and Glucose. Nanoscale 2016, 8 (6), 3685–3693. https://doi.org/10.1039/C5NR08038J.
19. Tian, R.; Sun, J.; Qi, Y.; Zhang, B.; Guo, S.; Zhao, M. Influence of VO2 Nanoparticle Morphology on the Colorimetric Assay of H2O2 and Glucose. Nanomaterials 2017, 7 (11), 347. https://doi.org/10.3390/nano7110347.
20. Ji, X.; Lu, Q.; Sun, X.; Zhao, L.; Zhang, Y.; Yao, J.; Zhang, X.; Zhao, H. Dual-Active Au@PNIPAm Nanozymes for Glucose Detection and Intracellular H2O2 Modulation. Langmuir 2022, 38 (26), 8077–8086. https://doi.org/10.1021/acs.langmuir.2c00911.
21. He, J.; He, D.; Yang, L.; Wu, G.-L.; Tian, J.; Liu, Y.; Wang, W. Preparation of Urchin-like Pd-PtIr Nanozymes and Their Application for the Detection of Ascorbic Acid and Hydrogen Peroxide. Mater. Lett. 2022, 314, 131851. https://doi.org/10.1016/j.matlet.2022.131851.
22. Lee, G.; Kim, C.; Kim, D.; Hong, C.; Kim, T.; Lee, M.; Lee, K. Multibranched Au–Ag–Pt Nanoparticle as a Nanozyme for the Colorimetric Assay of Hydrogen Peroxide and Glucose. ACS Omega 2022, 7 (45), 40973–40982. https://doi.org/10.1021/acsomega.2c04129.
23. Song, Y.; Qiao, J.; Liu, W.; Li Qi, L. Enhancement of gold nanoclusters-based peroxidase nanozymes for detection of tetracycline. Microchemical Journal 2020, 157, 104871.
24. Zhang, Z.; Tian, Y.; Huang, P.; Wu, F. Using target-specific aptamers to enhance the peroxidase-like activity of gold nanoclusters for colorimetric detection of tetracycline antibiotics. Talanta 2020, 208, 120342.

25. Chen, Y.; Xia, Y.; Yiwei Liu, Y.; Tang, Y.; Zhao, F.; Zeng, B. Colorimetric and electrochemical detection platforms for tetracycline based on surface molecularly imprinted polyionic liquid on Mn_3O_4 nanozyme. Biosensors and Bioelectronics 2022, 216, 114650.
26. Liu, B.; Zhu, H.; Feng, R.; Wang, M.; Hu, P.; Pan, J.; Niu, X. Facile molecular imprinting on magnetic nanozyme surface for highly selective colorimetric detection of tetracycline, Sensors and Actuators B: Chemical 2022, 370, 132451.
27. Wen, W.; Liu, Y.; Li, Z.; Wen, G.; Li, H.; Li, L. Magnetic Fe–N–C nanoparticles as a dual nanozyme for label-free colorimetric detection of antibiotics. Environ. Sci.: Adv., 2023,2, 731- 739.
